# Parliament and Publications

**Published:** 1911-04-22

**Authors:** 


### PARLIAMENT AND PUBLICATIONS.

### MILK AND TUBERCULOSIS.

The Board of Agriculture is considering the question of issuing an order requiring compulsory notification to a local authority, subject to a penalty for non-compliance, of all cases of milch cattle either affected with tuberculosis of the udder or visibly emaciated by tuberculosis.

### TUBERCULOSIS COMMISSION.

The final report of the Royal Commission on the relations between animal and bovine tuberculosis will be issued early in May, and the Appendices will follow soon afterwards. All experimental work finished over six months ago, and the Commission has been occupied since in drafting the Report.

### THE SALE OF POISONS.

In reply to Mr. Neville, who asked the Home Secretary whether he would consider the advisibility of making new regulations regarding the labelling of poisons, Mr. Churchill said no evidence had reached the Privy Council office that there is any pressing need for an amendment of the existing poison regulations in the direction indicated.

### PLAGUE INFECTION IN INDIA.

In reply to a question asked by Mr. Keir Hardie, Mr. Montagu said that exhaustive inquiry had shown that the plague bacillus does not live in the soil. It speedily dies outside the bodies of men or other animals. It is not, therefore, thought necessary to invite people to submit themselves to the inconvenience of evacuating their houses unless the disease is present or is imminent in a locality.

### OPIUM DENS IN LONDON.

In reply to a question asked in the House of Commons, Mr. Churchill said he was informed by the London County Council that there are known to the Council at the present time twenty-two private houses in London in which opium-smoking is practised, mostly by Chinese seamen. In 1906, the date of the issue of the Edict against opium-smoking in China, twelve such houses were known to the Council, but in the interval opium-smoking had been prohibited by the London County Council by-laws in seamen's lodging-houses licensed by them, and this had no doubt led to an increase in the number of private houses in which facilities for opium-smoking are afforded. The Commissioner of Police believes that opium-smoking is on the decrease in London.

### THE MEASLES EPIDEMIC.

A circular letter has been issued from the Local Government Board on the subject of the measles epidemic in London. The Board find that the School Attendance Department of the London Education Authority have supplied much valuable information as regards measles in the past, and the Board think that it might be practicable to arrange that this source of information should be made more readily available. It has now been arranged that so long as the present emergency lasts the school attendance officers will give speedy information to the medical officers of health of all cases of illness coming under their notice. It is hoped that, by this means, early information of the majority of cases of measles will reach the Medical Officers of Health. The Board have been in correspondence with the Metropolitan Asylums Board, and it is understood that the Board will agree to receive cases of measles on the recommendation of the Medical Officer of Health. The accommodation of the Metropolitan Asylums Board for this purpose is necessarily limited, and arrangements will be made by which preference will be given to those cases most needing hospital treatment.

### INSTRUCTION IN HYGIENE IN SCHOOLS.

In asking leave to introduce a Bill providing that simple instruction in hygiene shall be given in all public elementary schools, and to girls as to the feeding of infants, Dr. Addison said that in this country 150,000 children under the age of five die every year. He did not hesitate to say that at least 50,000 children die yearly as a direct or indirect consequence of parental ignorance. That hygienic instruction could be given with abundant success was now beyond the range of controversy. If we could obtain the incorporation of it into the code of education so much the better, and there would then be no need for this Bill. He had no desire to overcrowd the curriculum, but he thought many things, consisting chiefly of the mere recital of facts, far too long, might very well give place to something far more serviceable. The need for this instruction and training was urgent. By adopting it we should do very much, not only to improve the health of the children, but to bring them up in clean and wholesome habits and ideas, to stimulate and encourage their attention and alertness, and make them more useful in their day and generation, and give a better start in life to those who come after them.

